# A novel phage‐displayed MilA ELISA for detection of antibodies against *Myc. bovis* in bovine milk

**DOI:** 10.1111/jam.15655

**Published:** 2022-06-22

**Authors:** Mina Farzaneh, Abdollah Derakhshandeh, Abd Al‐Bar Ahmed Al‐Farha, Kiro Petrovski, Farhid Hemmatzadeh

**Affiliations:** ^1^ Department of Pathobiology School of Veterinary Medicine, Shiraz University Shiraz Iran; ^2^ Department of Animal Production Technical Agricultural College, Northern Technical University Mosul Iraq; ^3^ Australian Centre for Antimicrobial Resistance Ecology The University of Adelaide, School of Animal and Veterinary Sciences South Australia Australia; ^4^ Davies Research Centre School of Animal and Veterinary Sciences, The University of Adelaide Adelaide Australia; ^5^ School of Animal and Veterinary Sciences The University of Adelaide Adelaide Australia

**Keywords:** indirect ELISA, MilA antigen, Milk, *Myc. bovis*, phage display

## Abstract

**Aims:**

The aim of this study was to assess a phage‐displayed MilA protein of *Myc. bovis* in an indirect ELISA for the detection of *Myc. bovis* antibodies in milk samples.

**Methods and Results:**

The desired sequence of *milA* gene was synthesized and cloned into pCANTAB‐F12 phagemid vector. The expression of the MilA on the phage surface was confirmed by Western blotting. The recombinant phage was used in the development of an indirect ELISA to detect *Myc. bovis* antibodies in milk samples. There was a significant agreement between the results of phage‐based ELISA and recombinant GST‐MilA ELISA for the detection of *Myc. bovis* antibodies in milk samples.

**Conclusions:**

The inexpensive and convenient phage‐based ELISA can be used instead of recombinant protein/peptide ELISA as an initial screening of *Myc. bovis‐*associated mastitis.

**Significance and Impact of Study:**

Mastitis associated with *Myc. bovis* is a continuous and serious problem in the dairy industry. Sero‐monitoring of *Myc. bovis* infection cases are one of the key factors for surveillance of the infections in dairy farms. Despite the existence of some commercially serological assays for *Myc. bovis* antibodies, they have some limitations regarding their sensitivity and availability. The development of accurate diagnosis tools could contribute to control programmes of *Myc. bovis*‐associated mastitis in the dairy herds.

## INTRODUCTION

*Myc. bovis* is a bacterial pathogen associated with infection in cows of all ages (Al‐Farha et al., 2017; Petersen et al., 2020) and all over the world (Brank et al., 1999). *Myc. bovis*‐associated diseases such as arthritis, respiratory diseases and mastitis (Nicholas & Ayling, 2003; Stipkovits et al., 1999) cause serious economic losses in the cattle industry (Halasa et al., 2007; Parker, House, Hazelton, Bosward, Morton, & Sheehy, 2017; Petrovski et al., 2006). Antimicrobial resistance and superinfection with other pathogens make *Myc. bovis*‐associated diseases are difficult to treat. (Cai et al., 2005; Tenk et al., 2006). This pathogen is highly contagious and was first identified as the cause of mastitis in the United States in the 1960s (Calcutt et al., 2018). *Myc. bovis* intramammary infection may result in clinical or subclinical mastitis. Cows with subclinical infection are the main source of infection in dairy herds (Hazelton et al., 2018). Antibiotic treatment of *Myc. bovis‐*associated mastitis is often unsuccessful (Nicholas & Ayling, 2003). In the absence of an effective vaccine (Nicholas & Ayling, 2003; Stipkovits et al., 1999) and a cheap, rapid and accurate diagnostic method to detect mycoplasma mastitis (Al‐Farha et al., 2017; Ashraf et al., 2018), the development of the less expensive but effective diagnostic method for accurate screening of infected animals is an important tool for controlling the *Myc. bovis*‐associated mastitis (Le Grand et al., 2002). Bacterial isolation is the common method for the detection of *Myc. bovis*. The slow growth of this bacterium (Parker, House, Hazelton, Bosward, & Sheehy, 2017), intermittent secretion of bacteria (Hazelton et al., 2018), and exposure to antimicrobial treatment lead to false‐negative results in bacterial isolation (Caswell & Archambault, 2007). Nowadays, there is a great tendency to use alternative methods to detect *Myc. bovis* or host antibodies specific to it. These include conventional PCR, real‐time PCR, Loop‐mediated isothermal amplification (LAMP), enzyme‐linked immunosorbent assay (ELISA) and immunofluorescence assays (Al‐Farha et al., 2020). Using molecular and serological methods for the diagnosis of *Myc. bovis* could be affected by the availability, robustness and cost of the tests. High genetic and biochemical similarities between *Myc. bovis* and *Myc. agalactiae* is a challenge in identification using conventional bacteriological methods. Perhaps a combination of these methods would be the most effective in the diagnosis and control of the disease (Parker et al., 2018). To develop an effective serological test, it is important to find antigens with maximum specificity. Mycoplasma immunogenic lipase A protein (MilA) is a membrane protein with 303 kDa molecular weight. This protein acts as an auto transporter and adhesion component with additional lipase activity. Thus, this protein plays a complex role in the pathogenicity of *Myc. bovis* and stimulates the host immune responses (Adamu et al., 2020). Wawegama et al. (2014) and Adamu et al. (2020), reported that the N‐terminal region of the MilA with high immunogenicity, and low identity with the corresponding region of MilA from *Myc. agalactiae* (about 78% amino acid identity) making it an appropriate antigen for the development of specific serological tests (Adamu et al., 2020; Wawegama et al., 2014).

Several studies have demonstrated that the peptides or proteins displayed at the phage surface can be used as an antigen in antibody detection tests (Pan et al., 2015; Quanping et al., 2010; Wang et al., 2018; Yu et al., 2011). Phage display technology was introduced in 1985 (Smith, 1985). In this technique, the gene encoding the desired peptide is inserted into one of the protein coat genes, and subsequently, the desired peptide is fused to one of the phage‐coat proteins, and presented on the phage surface (Parmley & Smith, 1988; Scott & Smith, 1990; Wu et al., 2016). T4, M13 and lambda are bacteriophages that are generally used in this technique. Recently, M13 filamentous phage has been widely used for this purpose. Its genome contains a single‐strand DNA that encodes 11 different proteins including five coat proteins (Ledsgaard et al., 2018; Van Wezenbeek et al., 1980). pIII and pVIII with 406 and 50 amino acids, respectively, are the best capsid proteins for use in the phage display technology (Hamzeh‐Mivehroud et al., 2013). pIII is necessary for stabilization, infection and phage assembly (Hamzeh‐Mivehroud et al., 2013). This protein is used to display larger inserts (Fuh & Sidhu, 2000).

This study was aimed to express MilA peptide on the phage surface as a peptide fused to pIII protein and the use of this recombinant phage to generate an innovative ELISA for detection of antibodies against *Myc. bovis* in bovine milk samples.

## MATERIALS AND METHOD

### milA gene synthesis

To select the proper segment of the protein for expression, the available amino acid sequence (Uniprot accession: A0A454API1) was analysed in BioEdit software based on Kyte and Doolittle scale (Kyte & Doolittle, 1982). The part of the MilA protein sequence that showed the most hydrophilic properties was selected. For gene synthesis, the nucleotide sequences of *milA* gene (NCBI accession number YP_004056499) at position 1156 to 1659 was used. The selected sequence contained 504 nucleotides and was optimized for expression in *E. coli*. All of the TGA codons in the selected sequence were changed to TGG to match with *E. coli* expression. The PGEX‐4 T‐1 plasmid construct containing the desired sequence was synthesized (GenScript, West Lobby). The accuracy of the synthesized sequence was confirmed by Sanger sequencing.

### Construction of recombinant phagemid

The *milA* fragment was amplified in a PCR reaction using *Xba*I*milA*F primer (5′‐TCTAGAATGAGCGATAAATT AAATG‐3′), *Sal*I*milA*R primer (5′‐GTCGACATTCTTG AAAATGTTTA‐3′) and ALLTaq™ Master Mix PCR kit (Qiagen, Hilden, Germany). The PCR product was purified and sequenced in both directions to confirm the amplified insert fragment. pCANTABF12 phagemid used in this study was similar to pCANTAB5E but *Xba*I and *Sal*I cut sites were introduced into multiple cloning sites. The phagemid and *milA* amplified fragments were digested at 37°C with *Xba*I and *Sal*I‐HF restriction enzymes in CutSmart buffer (New England Biolabs. Inc.,). The gel‐purified products were ligated using Invitrogen™ T4 DNA ligase kit (Thermo Fisher Scientific) following the manufacturer's instruction. Ligation product was transfected to electrocompetent *E. coli* TG1 (Agilent) cells using the electroporation method described by Green and Sambrook (Green & Sambrook, 2012). The transformed colonies were screened for the presence of *milA*‐pCANTABF12 construct by colony PCR using vector‐specific primers (Baclioglu et al., 2014) and sequencing.

### Transfection and production of recombinant phage

The helper phage was amplified in *E. coli* XL1‐Blue MRF´ Strain (Agilent) in 2YT broth containing tetracycline. Phage titration was performed by the plaque counting method (Fahr & Frenzel, 2018). *E. coli* TG1 cells containing confirmed recombinant phagemid were cultured on a 2YT agar plate containing 100 μg ml^−1^ ampicillin (2YT‐A). Single colony was selected and transferred to 10 ml of 2xYT‐A broth medium. After overnight incubation, 2.5 ml of the medium was added to 500 ml of 2xYT‐A broth medium supplemented with 2% glucose and incubated (37°C, 250 RPM) until optical density of 600 nm reached the value of 0.6–0.7. At the next step, M13KO7 helper phage with the concentration of 10^10^ PFU ml^−1^ was added to the culture medium and incubated for 1 h without shacking, and an additional 1 h with shacking. The culture medium was centrifuged (3200 × g, 10 min, 4°C), the supernatant discarded and the pellet resuspended in 500 ml of fresh 2xYT broth medium supplemented with 0.1% glucose, ampicillin (100 μg ml^−1^), and kanamycin (50 μg ml^−1^). The culture was incubated overnight at 30°C in a shaker incubator as explained above. After overnight incubation, the culture was centrifuged twice (5000 × g for 20 min) and the supernatant was filtered through a 0.45 μm syringe filter. The resulted bacteriophages were precipitated using 20% polyethylene glycol in 2.5 mol l^−1^ sodium chloride and incubated at 4°C for at least 2 h. The bacteriophages were precipitated and washed two more times, each time being resuspended in one‐third of the initial volume (Baclioglu et al., 2014).

### SDS‐PAGE and Western blot

Expression of bacteriophage‐displayed antigen was checked in 12% SDS‐PAGE and visualized using silver staining method (Chevallet et al., 2006). BenchMark Pre‐Stained Protein Ladder (Life Technologies, Inc.,) was used to determine the protein size.

For Western blotting, recombinant and wild phages were run in polyacrylamide gel (same condition) and then transferred to PVDF (polyvinylidene difluoride) membrane by Trans‐Blot® Turbo™ RTA Mini PVDF Transfer Kit (Cat. No. 1704272; Bio‐rad). After transfer, the membrane was blocked using 10% BSA in PBS for 2 hours at room temperature under rotating. Anti‐E tag antibody (Abcam) in dilution 1:500 and anti‐mouse HRP antibody (Sigma‐Aldrich) in dilution 1:4000 were used as primary and secondary antibodies, respectively, and the membrane was developed using DAB tablet (3,3′‐diaminobenzidine tetra hydrochloride; Sigma‐Aldrich) dissolved in 15 ml of Tris‐buffered saline, pH 7.6 by adding 12 μl of fresh 30% hydrogen peroxide.

### Optimization of phage ELISA

An indirect ELISA was optimized to detect antibodies against *Myc. bovis* in bovine milk samples. Samples were taken from high somatic cell count cows aged 2–10 years without signs of clinical mastitis. Cows belonged to farms with a history of recurrent mastitis and high SCC that did not respond to antimicrobial treatment. The mean SCC value for tested milk samples was 378.04 (±SE = 60.49) × 10^3^ cells ml^−1^. Positive controls were selected from milk samples that were positive for *Myc. bovis* culture (Al‐Farha et al., 2017), qPCR and GST‐MilA ELISA (Al‐Farha et al., 2020). Negative controls were selected from milk samples that were negative for all three tests for *Myc. bovis* (Al‐Farha et al., 2017; Al‐Farha et al., 2020). GST‐MilA ELISA was developed by Wawegama et al. (2014) previously (Wawegama et al., 2014).

Initially, the optimal concentration of recombinant phage and milk sample (as a primary antibody) was determined by checkerboard titration (with seven dilutions of phage particles and three dilutions of positive and negative milk samples). The highest P/N ratio (OD_450_ of positive control milk/OD_450_ of negative control milk) was considered the optimal condition. Anti‐bovine IgG‐HRP (Sigma‐Aldrich) as secondary antibody was diluted 1:20,000, 1:40,000, 1:80,000, 1:160,000 and 1:320,000. Optical dilution of secondary antibody was found using optical concentration of phage particle and controls milk.

### Examination of collected milk samples

To assess the phage‐displayed antigen, 50 milk samples with known results for GST‐MilA ELISA and PCRs (Al‐Farha et al., 2020) were blindly tested in our phage‐displayed MilA‐ELISA. To evaluate milk samples, 100 μl of phage particles with a concentration of ~6 × 10^11^ PFU ml^−1^ diluted in the coating buffer (0.1 mol l^−1^ sodium carbonate and 0.1 mol l^−1^ sodium bicarbonate, pH 9.6) was added to each well of 96‐well Nunc MaxiSorp™ flat‐bottom ELISA plate (Thermo Scientific) and incubated overnight at 4°C. After overnight incubation, the plate was washed two times with 0.05% Tween‐20 (v/v) in PBS and then blocked using PBS containing 0.3 mmol l^−1^ BSA.

After 2 h incubation, the plate was washed two times. The milk samples were diluted 1 to 50 in the dilution buffer (PBS containing 0.15 mmol l^−1^ BSA and 0.05% (v/v) Tween‐20). 100 μl of dilution was added to each well and incubated for 2 h at room temperature (23–25°C). The ELISA plate was then washed four times and 100 μl of anti‐bovine HRP antibodies were diluted 1:20,000 in the dilution buffer, added to each well and re‐incubated for 1 h. After washing four times, the wells were developed using TMB (3,3′,5,5′‐tetramethylbenzidine) liquid substrate system for ELISA (Sigma–Aldrich, Inc.,) and incubated at RT for 5–15 min. Then, the reaction was stopped with sulphuric acid solution 2 mol l^−1^ and the optical density was read at 450 nm wavelength using a microplate reader (xMark™ Microplate Spectrophotometer, Bio‐Rad). Mean OD_450_ plus two standard deviation of negative milk samples was defined as the threshold value. Samples with optical density greater than threshold value were considered positive (Lardeux et al., 2016). We also examined the cross‐reactivity of bacteriophage surface proteins with milk samples utilizing M13KO7 helper phage as coating antigen.

The sensitivity and specificity of the phage‐based ELISA and GS‐MilA ELISA were compared with qPCR by the following formula:

Sensitivity = True Positive × 100/True Positive + False Negative.

Specificity = True Negative × 100/True Negative + False Positive.

### Data analysis

Statistical analysis was performed using SPSS version 16. The frequency of the agreement between GST‐MilA ELISA and phage‐based ELISA was measured by Cohen's kappa value. A value <0.20 mean poor agreement, a value between 0.21 and 0.40 mean fair agreement, a value between 0.41 and 0.60 mean moderate agreement, a value between 0.61 and 0.80 mean good agreement, and a value between 0.81 and 1.00 mean very good agreement (Altman, 1990).

## RESULTS

### Preparation of MilA‐Displaying phages

**FIGURE 1:**
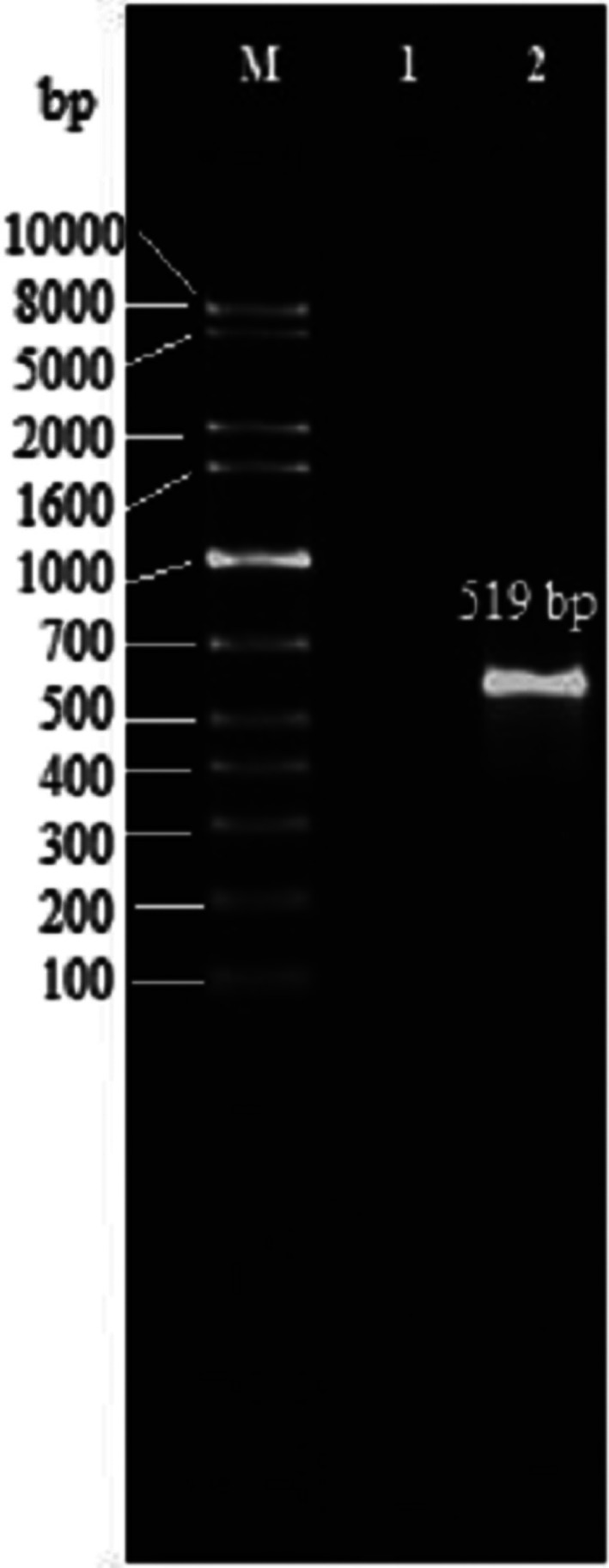
PCR amplification of *milA* fragment. Lane M: DNA marker (GelPilot 1 kb plus ladder [100], QIAGEN), lane 1: Negative control containing nuclease‐free water, lane 2: Amplified *milA* fragment (519 bp).

**FIGURE 2:**
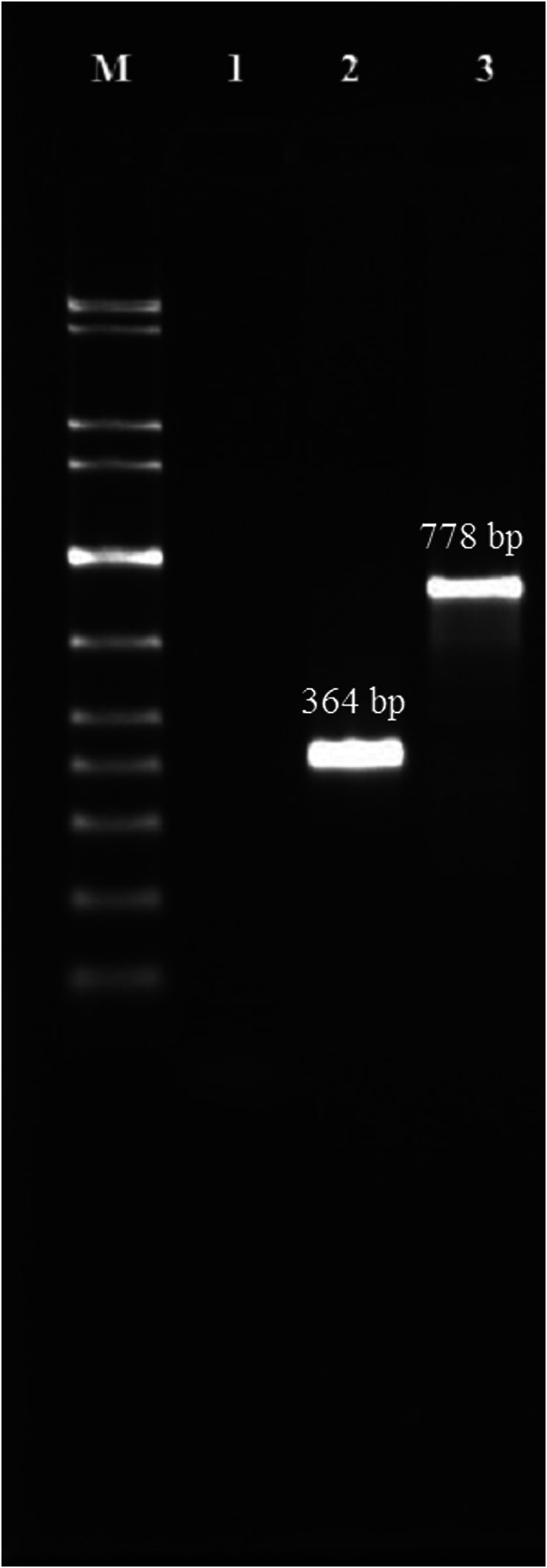
Colony PCR by vector‐specific primer. Lane M: DNA marker (GelPilot 1 kb plus ladder [100], QIAGEN), lane 1: Negative control containing nuclease‐free water, lane 2: Colony containing non‐recombinant pCANTABF12 showed 364 bp band on agarose gel, lane 3: Colony containing *milA*‐ pCANTABF12 construct showed 778 bp band on agarose gel.

The presence of a 519 bp band on agarose gel confirmed the target insert was successfully amplified in PCR (Figure 1). The digested insert using *XbaI* and *Sal*I‐HF restriction endonucleases was cloned into the pCANTABF12 phagemid vector. The positive recombinant clone was confirmed by colony PCR using vector‐specific primers. Colony containing non‐recombinant pCANTABF12 showed 364 bp band on agarose gel while colony containing *milA*‐pCANTABF12 construct showed 778 bp band (Figure 2). To verify the *milA*‐pCANTABF12 construct, the isolated phagemid from positive recombinant clone was sequenced using vector‐specific primers. Sequencing result showed the insert was in correct orientation. A single colony from pure culture and confirmed sequence were taken and used for further phage rescue process. Recombinant phage was rescued after superinfection of TG1 containing recombinant phagemid with M13KO7 helper phage.

### SDS‐PAGE and Western blot analysis of phage‐displayed peptide

**FIGURE 3:**
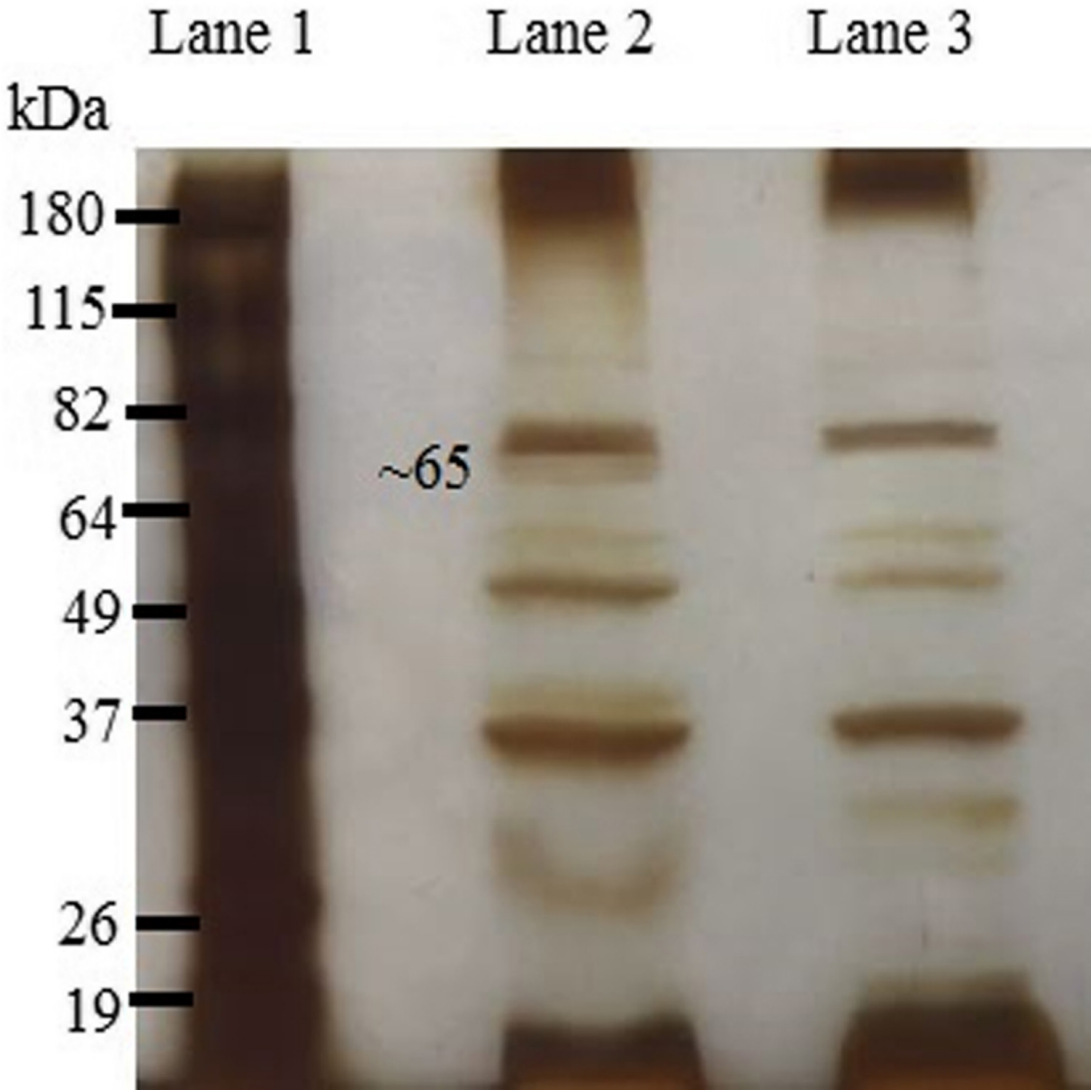
Silver staining SDS‐PAGE. Lane 1: Protein marker (BenchMark pre‐stained protein ladder (life technologies, Inc., Carlsbad, CA, USA)), lane 2: Recombinant phage displaying the MilA peptide (the presence of a band around 65 kDa indicated the expression of MilA on the phage surface), lane 3: Wild‐type phage.

**FIGURE 4:**
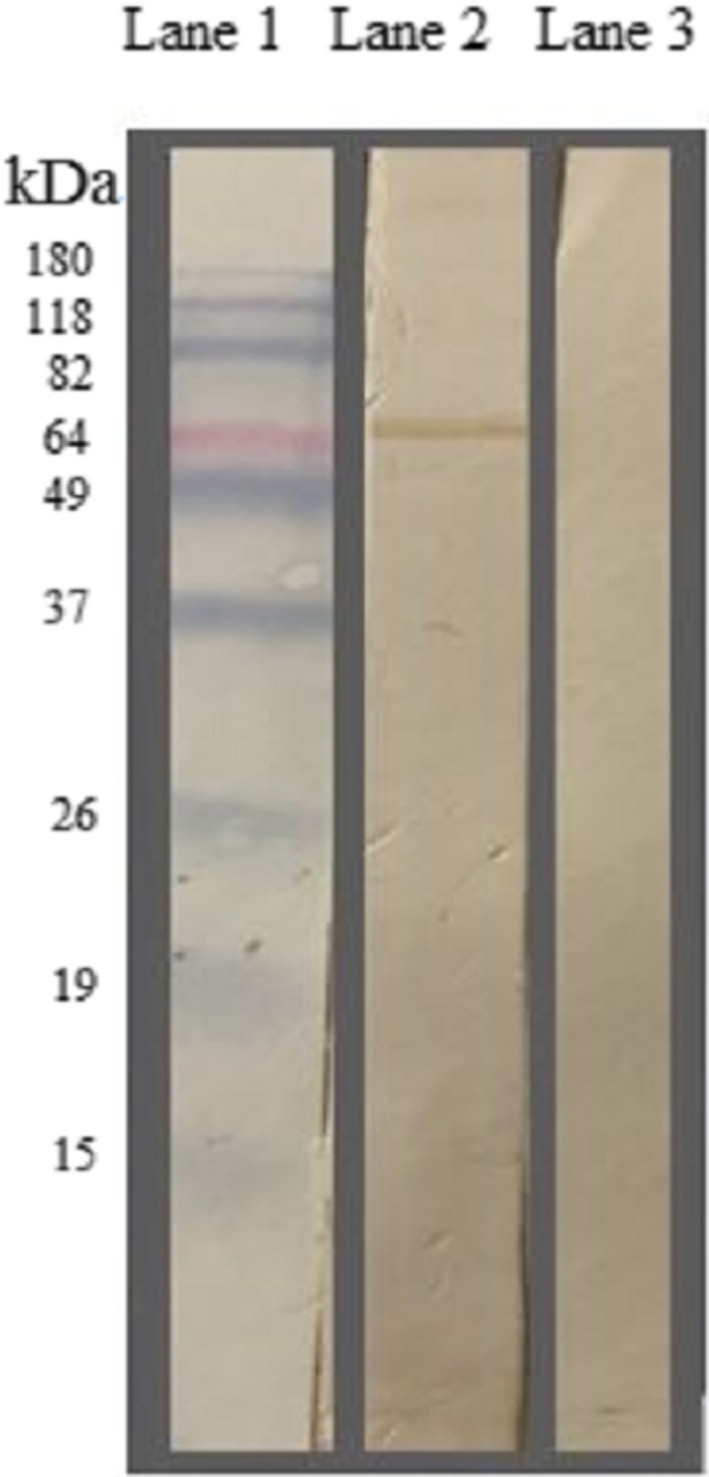
Western blot analysis of the recombinant MilA phage and wild‐type phages. Lane 1: Protein marker (BenchMark pre‐stained protein ladder (life technologies, Inc., Carlsbad, CA, USA)), lane 2: MilA recombinant phage displaying a band around 65 kDa: Probed with anti‐E tag antibody (Abcam, USA), lane 3: Wild‐type phage: Probed with anti‐E tag antibody (Lane 3).

SDS‐PAGE was performed to confirm the expression of antigen on the phage surface. The presence of a band around 65 kDa in running the recombinant phages and the absence of this band in wild phages indicated the expression of MilA on the phage surface (Figure 3). Observation of this band on a PVDF membrane in Western blot displayed peptide reacted successfully with the anti‐E tag antibody and demonstrated the accuracy of SDS‐PAGE results (Figure 4).

### Phage ELISA

The highest P/N ratio was obtained at milk dilution 1:50 and recombinant phage dilution 1:80 (~6 × 10^11^ PFU ml^−1^) (Table 1). The secondary antibody dilution 1:20,000 showed a stronger signal with less background in checkerboard ELISAs.

**TABLE 1:** Determination of the optimal concentration of recombinant phage and milk dilution based on P/N ratio (OD_450_ of positive control milk/ OD_450_ of negative control milk) by a checkerboard titration test

Phage dilution (first concentration: 5 × 10^13^ PFU ml^−1^)	OD_450_ ratios between positive control milk and negative control milk (P/N ratio)		
	Milk dilution		
	1:25	1:50	1:100
1:10	4.83	5.47	5.23
1:20	4.93	5.48	5.19
1:40	5.52	6.16	5.17
1:80	5.35	6.22	5.68
1:160	4.92	5.84	5.21
1:320	4.16	5.28	4.97
1:640	3.49	4.24	3.82

### Detection of anti‐MilA antibodies in milk samples by phage ELISA

All samples were previously checked by GST‐MilA ELISA (Al‐Farha et al., 2020), in this study, they were analysed using the phage‐MilA ELISA (Table 2). When a threshold value of 0.35 was used 10 samples of 50 samples showed positive results in phage‐based ELISA for *Myc. bovis* antibodies detection. Of the 8 positive samples on the GST‐MilA ELISA, 7 tested positive and one was negative to the phage‐based ELISA. Of the 42 negative samples on the GST‐MilA ELISA, 39 tested negative and 3 positive to the phage‐based ELISA. The sensitivity and specificity of the phage‐based ELISA were 59% and 90%, respectively, whilst the sensitivity and specificity of the GST‐MilA ELISA were 56% and 90%, respectively (Table 3). The result of statistical analysis showed a good agreement in the detection of *Myc. bovis* antibodies in milk samples between the GST‐MilA ELISA and the phage‐based ELISA (Cohen's kappa coefficient, 0.73 [95% CI, 0.6–0.86]). Furthermore, no reactions were observed to the bacteriophage surface proteins in the tested milk.

**TABLE 2:** Comparison of phage‐based ELISA result with GST‐MilA ELISA in detecting *Myc. bovis* positive milk samples

	GST‐MilA ELISA results (no.)		Total
	Negative	Positive	
Phage‐based ELISA results (no.)			
Negative	39	1	40
Positive	3	7	10
Total	42	8	50

**TABLE 3:** The outcome of the phage‐based ELISA and GST‐MilA ELISA in comparison to qPCR. qPCR results obtained from Al‐Farha et al. (2020)

qPCR results (no.)	GST‐MilA ELISA results (no.)			Phage‐based ELISA results (no.)		
	Positive	Negative	Total	Positive	Negative	Total
Positive	5	18	23	7	16	23
Negative	3	24	27	3	24	27
Total	8	42	50	10	40	50

## DISCUSSION

The current study was conducted to evaluate a novel phage‐based ELISA in its capability to detect antibodies against *Myc. bovis* in bovine milk samples. The preliminary results demonstrated that phage‐ELISA can detect anti‐MilA antibodies in milk samples. It also showed that the recombinant MilA phage at the dilution of ~6 × 10^11^ PFU ml^−1^ contains sufficient amount of antigen for detection of anti‐MilA antibodies in ELISA. Although the number of tested samples was limited (*n* = 50), the inclusion of appropriate positive and negative controls in the test ensures results could be applicable to the external population. The phage‐displayed MilA antigen has some advantages compared to the recombinant MilA protein production. The purification process for recombinant proteins contains complicated steps of affinity purification, protease treatment for removing the fusion tags. It makes the recombinant proteins more expensive, time‐consuming and hard to make in uniform batches (Du & Rehm, 2017). The large‐scale production of the phage‐displayed antigens is less expensive, more uniform and easier than other methods (Baclioglu et al., 2014). Additionally, phage‐displayed antigens are more resistant to tough environmental conditions and keep their uniformity for longer time compared to the bacterially expressed recombinant proteins (Aghebati‐Maleki et al., 2016). Regarding all above advantages, using the phage‐displayed MilA antigen is recommended in MilA ELISA to assess the *Myc. bovis* situation in dairy farms.

To date, different recombinant antigens have been produced by expression in *E. coli* and evaluated for detection of *Myc. bovis* antibody in ELISA. Variable surface proteins are highly immunogenic antigens, but phase variation by switching their expression OFF or ON and size variation caused variable antibody response against them (Brank et al., 1999). PDHB is a conserved and immunogenic protein, but PDHB‐based ELISA has shown cross‐reaction with *Myc. agalactiae* (Sun et al., 2014). rMbovP579‐based ELISA showed high sensitivity (90.2%) and specificity (97.8%) (Khan et al., 2016). Furthermore, direct competitive ELISA to detect P48 protein without cross‐reactivity with other bacteria has shown higher positive detection rates than Biovet and Bio‐X ELISA kits (Fu et al., 2014). MilA is an immunogenic protein that is not affected by phase or antigenic variation (Wawegama et al., 2014). An indirect IgG ELISA was developed based on MilA‐protein that showed high specificity (98.7%) and sensitivity (92.86%) (higher than Bio‐X ELISA kits) (Wawegama et al., 2014; Wawegama et al., 2016). Previous studies have shown MilA ELISA can detect antibodies against *Myc. bovis* from calves aged 3 weeks and older (Petersen et al., 2018), and it could be applied to screen cows before joining a naive herd or in a systematic programme aimed to have a herd free of *Myc. bovis* infection (Wawegama et al., 2016). Also, GST‐MilA ELISA can be used for the detection of anti‐MilA antibodies in bovine milk samples (Al‐Farha et al., 2020). Several commercial kits are available for sero‐monitoring of *Myc. bovis* infections in cows including, Bio‐X Diagnostics (Rochefort, Belgium), Biovet Inc (Quebec, Canada) and ID screen® *Myc. bovis* indirect ELISA (IDvet (Grabels, France)). These kits are not yet accessible in some countries. The Sensitivity of the IDvet ELISA and the BIO K302 ELISA for detection of *Myc. bovis* antibodies in serum sample have been reported, 93.5% and 49.1%, respectively, and the specificity has been shown 98.6% and 89.6%, respectively (Andersson et al., 2019).

This study showed the sensitivity of phage‐based ELISA was a bit higher than that of GST‐MilA ELISA. It is possible that the correct conformation of peptides on the phage surface leads to better recognition of these peptides in the phage‐based ELISA (Portefaix et al., 2002). Moreover, we used the most hydrophilic part of the MilA peptide that showed the highest immune response to *Myc. bovis*‐specific calf sera and was used to optimize the GST‐MilA ELISA. (Wawegama et al., 2014). The hydrophilic region increases the water solubility and surface exposure of antigen (Berzofsky, 1985). It is important to state that both ELISAs showed much lower sensitivity compared to the qPCR. Determining the sensitivity and specificity of ELISA compared to the qPCR is complex and not without limitations. The qPCR detects the presence of *Myc. bovis* DNA whilst indirect ELISA detects *Myc. bovis* antibodies. There was no obligation that either *Myc. bovis* bacteria or antibodies against *Myc. bovis* existed in the milk samples at the time of the experiment. Thus, it is our opinion that ELISA may be appropriate for initial screening, followed by qPCR for verifying the active presence of *Myc. bovis* in the samples.

Several studies have revealed the potential use of the phage‐based ELISA to antibody detection. Quanping et al. (2010), reported that despite the lower sensitivity of ELISA with hybrid phage compared to ELISA with recombinant protein, there was no significant difference between these methods for *Candida albicans* diagnosis (Quanping et al., 2010). Another report showed the detection rate of *C. albicans* in ELISA with Sap2 and Hsp90 hybrid phages was higher than in ELISA with Sap2 and Hsp90 recombinant protein (Wang et al., 2018). Yu et al. (2011), showed that p53 phage‐based ELISA in comparison with recombinant p53 ELISA showed lower sensitivity but higher specificity in carcinoma‐antibody detection. They noted that the decrease in the phage‐based ELISA sensitivity may be due to the use of small peptides for phage display (Yu et al., 2011). Pan et al. (2015), reported that p53 hybrid phage‐based ELISA for detection of anti‐p53 antibody in patients with a malignant tumour had a lower detection rate than ELISA with recombinant protein, but using both recombinant protein and hybrid phage increased the detection rate (Pan et al., 2015).

In conclusion, this study has shown good agreement between the phage‐based ELISA and GST‐MilA ELISA. Due to the advantages of the phage display technology, the phage‐based ELISA rather than the recombinant protein/peptide ELISA could be used as an initial screening of *Myc. bovis‐*associated mastitis. This is supported by the cost‐effectiveness in the production of the phage‐displayed antigen. Indeed, the novel ELISA proposed in this study requires comparison to commercial ELISA kits that have shown high sensitivity and specificity.

## CONFLICT OF INTEREST

No conflict of interest declared.
